# Reduction of Femoral Fractures in Long-Term Care Facilities: The Bavarian Fracture Prevention Study

**DOI:** 10.1371/journal.pone.0024311

**Published:** 2011-08-30

**Authors:** Clemens Becker, Ian D. Cameron, Jochen Klenk, Ulrich Lindemann, Sven Heinrich, Hans-Helmut König, Kilian Rapp

**Affiliations:** 1 Department of Clinical Gerontology, Robert-Bosch-Hospital, Stuttgart, Germany; 2 Rehabilitation Studies Unit, Sydney Medical School, University of Sydney, Ryde, Australia; 3 Institute of Epidemiology and Medical Biometry, Ulm University, Ulm, Germany; 4 Department of Medical Sociology and Health Economics, University Medical Center Hamburg-Eppendorf, Hamburg, Germany; Federal University of Rio de Janeiro, Brazil

## Abstract

**Background:**

Hip fractures are a major public health burden. In industrialized countries about 20% of all femoral fractures occur in care dependent persons living in nursing care and assisted living facilities. Preventive strategies for these groups are needed as the access to medical services differs from independent home dwelling older persons at risk of osteoporotic fractures. It was the objective of the study to evaluate the effect of a fall and fracture prevention program on the incidence of femoral fracture in nursing homes in Bavaria, Germany.

**Methods:**

In a translational intervention study a fall prevention program was introduced in 256 nursing homes with 13,653 residents. The control group consisted of 893 nursing homes with 31,668 residents. The intervention consisted of staff education on fall and fracture prevention strategies, progressive strength and balance training, and on institutional advice on environmental adaptations. Incident femoral fractures served as outcome measure.

**Results:**

In the years before the intervention risk of a femoral fracture did not differ between the intervention group (IG) and control group (CG). During the one-year intervention period femoral fracture rates were 33.6 (IG) and 41.0/1000 person years (CG), respectively. The adjusted relative risk of a femoral fracture was 0.82 (95% CI 0.72-0.93) in residents exposed to the fall and fracture prevention program compared to residents from CG.

**Conclusions:**

The state-wide dissemination of a multi-factorial fall and fracture prevention program was able to reduce femoral fractures in residents of nursing homes.

## Introduction

The incidence of falls in nursing homes is about three times that in the community and is equivalent to approximately 1.5 falls per bed per year [1]. Femoral fractures are one of the most important and frequent fall related injury in this group. In industrialized countries 20% to 30% of femoral fractures occur in nursing care facilities [2] and the rate of femoral fractures in institutions is about 10 times that in the community [3]. Up to now, community living older people have been the main target group of preventive efforts [4]. Pharmaceutical approaches targeting bone health have been advocated [5]. Some studies have included exercise and other non-pharmaceutical interventions [6] to reduce falls and fall related injuries. There is evidence that fall prevention interventions reduce falls [6] but there is little evidence to support a reduction in fractures [7]; [8].

In 2003 we published first results of a successful fall prevention program in long-term care [9]. As a consequence first large translational effort was conducted in south-western German long-term care facilities in one state during 2003–2004. The results on femoral fracture reduction rates in more than 170 facilities compared with a control group were inconclusive [10]. The interventions partly relied on recommendations and referral that could not be influenced by the staff members responsible for the implementation. The nursing homes participated on a voluntary basis without making a signed agreement. The hip protector usage was not supported.

The implementation process was therefore redesigned and we planned a second large scale effort in the German state of Bavaria. The project aimed to introduce a fracture and fall prevention program that combined a reduction of risk factors such as exercise or medication modification together with hazard compensation components, for example the recommendation of hip protectors and environmental modifications.

The goal of the project was to reduce the femoral fracture rate in the participating institutions within one year of participation.

## Methods

### Intervention program

In 2007 Germany's largest health insurance company (AOK) decided to fund the implementation of *The Bavarian Fall and Fracture Prevention Study* in 256 nursing homes in Bavaria, Germany. The health care fund had no influence on the intervention components but participated in the implementation process. The evaluation of the intervention was not funded and not influenced by the AOK as well. The components of the interventions were modified from a previous randomized controlled study [9] and followed a manual [11]. Participation in the program was voluntary for each institution and there were no sanctions on nursing homes that did not participate. Participating nursing homes had to sign a written contract to improve uptake and adherence including the participation in a standardized benchmarking system to document falls and fall related injuries. The fall prevention program was offered to all residents of participating nursing homes independently from membership to a specific health insurance company. For the analyses reported in this paper, however, data were only available from residents insured at the AOK. The AOK covers nearly 50% of residents living in nursing homes in Bavaria.

The program is summarized in Table 1. It included the teaching of change agents and exercise instructors. The change agents, mostly senior nursing staff members, were asked to disseminate the knowledge on fall and fracture prevention within the homes. The key messages were the feasibility of fall prevention to empower and engage nursing staff and assistants as well as general physicians and therapists. On the nursing home level they were encouraged to look for person-environment mismatches using an environmental check list but focusing on small adaptations like bed height, grab bars or proper lighting. Eligible residents were offered participation in an exercise program. The amount of exercise per week was doubled from a previous translational effort [10] to reach an appropriate frequency. The exercise program consisted of progressive strength and balance training which was delivered in groups of 8 to 10 residents (1 hour twice a week). This doubles the dose of the previous intervention.

**Table 1:** Components and details of the intervention program.

Component	Details
Exercise	Progressive strength and balance training; 1 hour twice a week; groups of 8–10 participants; to qualify for exercise groups, residents had to be able to stand with support; exercise instructors for the first 6 months were physiotherapists or sport therapists, supported by a member of the nursing home staff; after 6 months the training was taken over by members of the nursing home staff
Documentation of falls	Compulsory; documentation sheets were sent to the health care insurance; regular feedback on fall statistics
Environmental adaptations	Nurses were encouraged to look for person-environment mismatches using an environmental check list which included more than 100 items [19]
Medication review, vitamin D	Nurses were encouraged to discuss a regular medication review with the physicians focusing on reduction of inappropriate psychotropic drugs, and the prescription of vitamin D.
Hip protectors	Each home received a test kit of 5 hip protectors for demonstration purposes; recommendation of hip protectors was part of the program but they were not reimbursed by most German health care insurance companies.
Education and education materials	Change agents received a one-day training course; exercise instructors received a different one-day training course; manual with all contents of the program [11]; material for in-house education; web page with additional information [20]

The change agents were encouraged to discuss a regular medication review with the physicians focusing on inappropriate psychotropic drugs and the prescription of vitamin D. The individual use of hip protectors was recommended by the project but not reimbursed. Differing from the previous translational project [10], each home received a test kit of 5 hip protectors for demonstration purposes. Aside from the implementation manual [11], a demonstration package was available that contained a video on the intervention, leaflets, and material for in-house teaching and family caregiver information. The institutions were encouraged to market the program as state-of-the-art and to gain public recognition and media coverage where ever possible. This was supported by the health insurance fund. All institutions were regularly visited by staff of the regional offices of the health insurance fund who were trained to support the project.

Recording of the falls and injuries for all residents was compulsory and started 3 months prior to implementation of the intervention (January to March 2007). This was done to increase awareness of all staff members and the receptiveness of the institutions. Regular feedback on fall statistics was provided to each participating institution. The data included comments if the documentation was inappropriate.

The change agents from each participating nursing home initially received a one-day training course in the program. The training sessions for the physiotherapists were also limited to a one day course. The intervention period started at 1st April 2007 and continued for 12 months. The study was approved by the ethical committee of Ulm University. Only routine data which were already available at the AOK for other purposes were used for this analysis. Therefore, no informed consent was necessary. The AOK gave permission to reanalyse and publish the data.

### Level of care

To newly enter into a nursing home in Germany, residents must fulfil the requirements of the mandatory long-term care insurance. This insurance was introduced in 1995. The insurance is compulsory for all citizens [12]. New residents must have a certain level of ADL dependency. Depending on the amount of care required, recipients are categorized into one of three levels after an assessment by a physician (level 1, 2, and 3 requiring basic care such as washing, feeding, or dressing for at least 0.75, 2 and 4 hours per day, respectively). More than 50% of the residents have significant disability due to dementia. Other important groups are chronic stroke survivors and residents receiving palliative care.

### Outcome

Incident femoral fractures (ICD-10: S72) were the primary outcome. Since fall documentation was an important part of the intervention program incident falls were only available from residents of intervention homes. A comparison between fall rates of the intervention homes and control homes was therefore not possible. The absolute number of falls in the 3 months before the start of the intervention and 3 to 6 months after the start of the intervention was compared for those homes included in the intervention group.

### Data source

Routine data collection systems of the health insurance company were utilized to obtain data on gender, age, date of admission to the home, level of care (see below) and, if appropriate, femoral fractures and date of death for each individual. Hospital discharge diagnoses were used to identify femoral fractures.

### Statistics

Residents aged 65 years and more, insured at the AOK, assigned to a level of care and living between 1 April 2007 and 31 March 2008 in a Bavarian nursing home were included in the main analysis. Control homes were those homes not included in the program in 2007 and not intending to start with the program in 2008.

The incidence rate of femoral fractures was calculated by dividing the number of fractures by the total number of person-years. Proportional hazard regression models were applied. Intervention status was the independent variable, and time to femoral fracture or time to censoring the dependent variable. The regression models were adjusted for gender, age, level of care and size of the nursing home (logarithm of the number of beds).

Since nursing homes were not randomised the selection of homes may have influenced the outcome. Therefore, femoral fracture rates were also calculated for the preceding years (2001, 2002, 2003, 2004, 2005, and 2006). For each calendar year fracture rates were compared between the homes assigned to be in the intervention group in 2007 and the homes assigned to be in the control group in 2007. Only data from nursing homes were used which were included in the main analysis for 2007.

## Results

The fall prevention program was introduced in 256 nursing homes with 13,653 residents.

The control group consisted of 893 nursing homes with 31,668 residents. Age, gender and level of care were comparable between residents of the intervention group and the control group. Participating facilities were larger than non-participatory facilities (Table 2).

**Table 2:** Characteristics of the study population institutionalized in Bavarian nursing homes during the first year of intervention (1 April 2007-31 March 2008).

	Intervention group	Control group
Nursing homes		
N	256	893
Number of beds		
Mean (SD)	94.4 (41.3)	70.8 (43.7)
Study population		
Gender		
Male, n (%)	2,892 (21.2)	6,828 (21.6)
Female, n (%)	10,761 (78.8)	24,840 (78.4)
Age (years)		
Mean (SD)	84.3 (7.5)	84.2 (7.7)
Level of care, n (%)		
1	5,271 (38.6)	12,175 (38.5)
2	5,375 (39.4)	12,748 (40.3)
3	3,007 (22.0)	6,745 (21.3)

During the one-year intervention period femoral fracture rates were 33.6/1000 person-years in the intervention homes and 41.0/1000 person-years in the control homes resulting in an 18% reduction of femoral fractures in residents from intervention homes in the multivariate analysis (hazard rate ratio 0.82, 95% confidence interval 0.72–0.93) (Table 3). In absolute terms this is approximately equivalent to a reduction of 1.5 hip fractures each two years in a 100 bed nursing home. The effect was somewhat stronger in women (HR 0.80, 95% CI 0.69–0.92) than in men (HR 0.93, 95% CI 0.69–1.27) (p for interaction 0.23).

**Table 3:** Effect of the fall prevention program on femoral fracture incidence in Bavarian nursing homes during the first year of intervention (1.04.2007–31.03.2008).

	Femoral fractures, n	Total person-years	Femoral fractures/1000 person-years	HR (95% CI)*
Fall prevention program				
No (Control group)	917	22,450	41.0	1.00
Yes (Intervention group)	331	9,882	33.6	0.82 (0.72–0.93)

*Hazard rate ratio and 95% confidence interval adjusted for gender, age, number of beds (log) and level of care.

**Figure 1:**
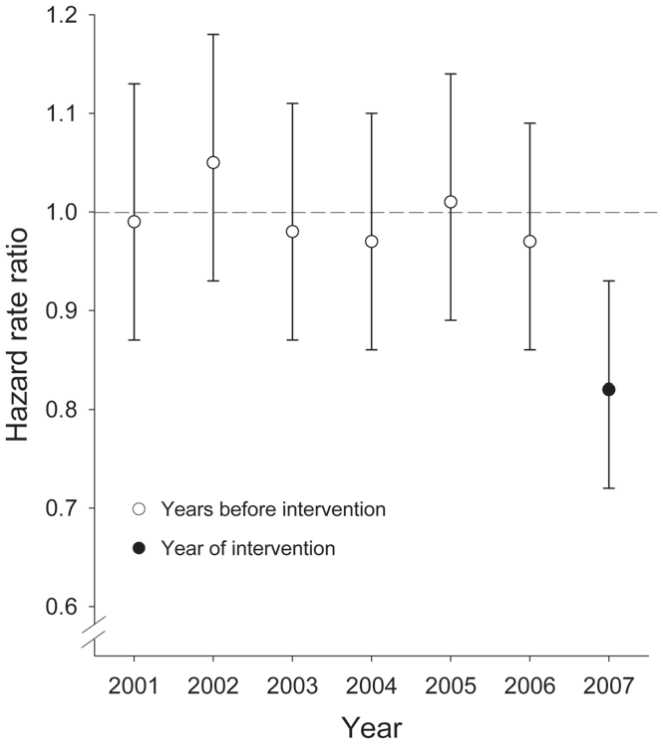
Relative risk of femoral fractures between residents from intervention and control homes in the years before the start of the intervention (2001–2006) and in the year of the intervention (2007).

Femoral fracture rates in residents from intervention and control homes were similar in the years before the start of the intervention (e.g. 40.0 and 41.2/1000 person-years in 2004; 38.7 and 38.8/1000 person-years in 2005; 37.9 and 39.3/1000 person-years in 2006). Figure 1 shows the relative risk of femoral fractures between residents from intervention and control homes. The risk is close to 1 in the years before the start of the intervention (2001–2006) and drops to 0.82 in the year after the implementation of the intervention (2007).

Comparing the 3 months before the start of the intervention and the months 3 to 6 after the start of the intervention a reduction of the number of falls by 8.3% was observed.

## Discussion

The evaluation of this translational study demonstrates for the first time that it was feasible to reduce femoral fractures in long-term care residents with an adequate sample size. This is an important outcome. The rate of misascertainment is minimal since almost all people with femoral fractures are admitted to hospital.

It has been often criticized that the knowledge we have from randomized controlled trials has not been disseminated into daily life [13]; [14]. This study is an example that a large-scale translation of an evidence-based program into daily practice can be successful if it is supported by policy or influential stakeholders of the health care system such as a large health care insurance company. The strength of such a translation study is its power which allows analysis of relatively rare endpoints like femoral fractures. In our analysis more than 40,000 participants from more than 1,000 institutions were included. The disadvantage is its study design since the homes were not randomized and the intervention group may have been a selection of nursing homes with lower fracture rates. The intervention and control groups, however, had very similar femoral fracture rates over several years before the project started. This supports that our major finding, the reduction of femoral fractures in the intervention year, is actually caused by the intervention. We were unable to identify a confounding factor other than the fall and fracture prevention program that would have reduced femoral fractures only in the intervention nursing homes. However, this cannot be fully excluded.

An important question is why the program was successful and a project from our group conducted in 2003–4 failed to demonstrate similar results [10]. There are a number of components within the implementation process that were redesigned. The changes partly related to the intervention components themselves, and even more important to the process of education and the way the program was implemented. These might have improved the uptake and adherence by the institutions. In the current project in Bavaria the participating nursing homes had to increase their commitment by signing a contract to participate. A national nursing guideline on fall prevention in long-term care was published after the first study in 2004. This increased the receptiveness of the institutions. The facilities were invited to use the intervention as a “gold standard” to fulfil the requirements of the guideline. Other factors were the introduction of changes to training, and implementation and monitoring of the intervention. The educational process was more interactive. The institutions received several visits. Improved positive media coverage was sought about the role of exercise in long-term care facilities. Questions of liability were addressed by pointing out that the participating homes would be considered as state-of-the-art facilities thus decreasing the likelihood of litigation. This aspect was reinforced by a decision of the Federal Court of Justice in 2005 that fall prevention must be offered to residents in long term care.

No process evaluation was performed in the intervention homes because a close evaluation may have changed behaviour in the homes under observation. However, participation in the exercise program, and availability and use of hip protectors, were evaluated in about 4,000 residents of 48 nursing homes which started with the same intervention program one year later. The participation rate in the strength and balance training differed considerably between nursing homes (median 13.5%, range 3.4 to 47.8%). The prevalence of hip protectors was 10.0% in women and 6.2% in men. 64% of residents with a hip protector used it during the four weeks prior to the examination. Again, there was a large variability in the prevalence of use of hip protectors between nursing homes [15]; [16].

Even though the rate of femoral fractures decreased we believe that more fractures can be prevented. The interaction between physicians and nursing staff is often suboptimal and, as a result, medication review [17] and vitamin D supplementation [6] are still underused. We recently published data on fracture rates of newly admitted nursing home residents. We observed that the risk of a fracture was highest during the first months after admission and declined thereafter [18]. It is therefore a challenge to improve nursing processes during this initial period. As discussed above participation rates in the exercise program and prevalence of hip protectors differ considerably between the different nursing homes and are therefore also targets for future improvements. New approaches with real-fall analysis including video data and accelerometers might further improve our understanding of falls and fractures.

In conclusion, the Bavarian fracture prevention study demonstrates positive results of a multifactorial program for fracture prevention. After the first study year the program has now been introduced in more than 2,000 facilities in Bavaria and other German states, and neighbouring countries. The evaluation will continue to examine sustainability and cost-benefit results. First results are expected in 2012.

## References

[1] Rubenstein LZ, Josephson KR, Robbins AS (1994). Falls in the nursing home,. Ann Intern Med.

[2] Pocock NA, Culton NL, Harris ND (1999). The potential effect on hip fracture incidence of mass screening for osteoporosis,. Med J Aust.

[3] Butler M, Norton R, Lee-Joe T, Cheng A, Campbell AJ (1996). The risks of hip fracture in older people from private homes and institutions,. Age Ageing.

[4] Gillespie LD, Robertson MC, Gillespie WJ, Lamb SE, Gates S (2009). Interventions for preventing falls in older people living in the community,. Cochrane Database Syst Rev.

[5] Chapuy MC, Arlot ME, Duboeuf F, Brun J, Crouzet B (1992). Vitamin D3 and calcium to prevent hip fractures in the elderly women,. N Engl J Med.

[6] Cameron ID, Murray GR, Gillespie LD, Robertson MC, Hill KD (2010). Interventions for preventing falls in older people in nursing care facilities and hospitals,. Cochrane Database Syst Rev.

[7] Ward JA, Harden M, Gibson RE, Byles JE (2010). A cluster randomised controlled trial to prevent injury due to falls in a residential aged care population,. Med. J Aust.

[8] Cox H, Puffer S, Morton V, Cooper C, Hodson J (2008). Educating nursing home staff on fracture prevention: a cluster randomised trial,. Age Ageing.

[9] Becker C, Kron M, Lindemann U, Sturm E, Eichner B (2003). Effectiveness of a multifaceted intervention on falls in nursing home residents,. J Am Geriatr Soc.

[10] Rapp K, Lamb SE, Erhardt-Beer L, Lindemann U, Rissmann U (2010). Effect of a statewide fall prevention program on incidence of femoral fractures in residents of long-term care facilities,. J Am Geriatr Soc.

[11] Becker C, Lindemann U, Rissmann U, Warnke A (2006). Sturzprophylaxe. Sturzgefährdung und Sturzverhütung in Heimen, Vincentz Network Hannover.. ISBN.

[12] Becker C, Leistner K, Nikolaus T, in: Michel JP, Rubenstein LZ, Vellas BJ, Albarede JL (1998). Introducing a statutory insurance system for long-term care (Pflegeversicherung) in Germany.

[13] Glasgow RE, Emmons KM (2007). How can we increase translation of research into practice?. Types of evidence needed, Annu.Rev.Public Health.

[14] Glasgow RE (2008). What types of evidence are most needed to advance behavioral medicine?. Ann.Behav.Med..

[15] Becker C, Rapp K (2010). Fall prevention in nursing homes, Clin.Geriatr Med..

[16] Klenk J, Kurrle S, Rissmann U, Kleiner A, Heinrich S (2011). Availability and use of hip protectors in residents of nursing homes,. Osteoporos.Int. Osteoporos Int..

[17] Zermansky AG, Alldred DP, Petty DR, Raynor DK, Freemantle N (2006). Clinical medication review by a pharmacist of elderly people living in care homes–randomised controlled trial,. Age Ageing.

[18] Rapp K, Lamb SE, Klenk J, Kleiner A, Heinrich S (2009). Fractures after nursing home admission: incidence and potential consequences,. Osteoporos Int.

[19] Marx L, Nußberger J, Ziller A (1994). Barrierefreie Wohnungen, Leitfaden zu den Planungsgrundlagen..

[20] Robert-Bosch-Hospital Stuttgart http://www.aktiv-in-jedem-alter.de.

